# Evaluation of Possible Toxic Effects of Boric Acid in Palourde Clam (*Ruditapes decussatus*) Through Histological Changes and Oxidative Responses

**DOI:** 10.1007/s12011-024-04230-4

**Published:** 2024-05-14

**Authors:** Selin Ertürk Gürkan, Mert Gürkan, Volkan Sarıtunç, Ezgi Can İbiş, Berkay Güneş

**Affiliations:** 1 Department of Biology, Faculty of Science, Çanakkale Onsekiz Mart University, Çanakkale, Turkey; 2 School of Graduate Studies, Çanakkale Onsekiz Mart University, Çanakkale, Turkey

**Keywords:** Boron derivatives, Oxidative stres, Histopathology, Bivalve

## Abstract

The extensive utilization of boric acid, particularly in industrial and agricultural sectors, also engenders concerns regarding the toxicity of boron and its derivatives. Particularly, the behavior of boric acid at increasing concentrations in aquatic ecosystems remains poorly understood. In light of these concerns, this study aimed to investigate the toxicity of boric acid in bivalves, which occupy a critical position in the food chain. Specimens of *Ruditapes decussatus*, which had not been previously exposed to any pollutants and were cultivated under controlled conditions, were subjected to three different concentrations of boric acid (0.05 mg/L, 0.5 mg/L, and 5 mg/L) in vitro for 96 h. Following the exposure period, the specimens were assessed for histological changes (the mantle, gill, and digestive gland) and specific oxidative parameters (the gill and digestive gland), including superoxide dismutase (SOD), catalase (CAT), glutathione-S-transferase, and lipid peroxidation (LPO). The research findings indicated that boric acid primarily induced oxidative damage at the applied concentrations and increased antioxidant levels (*p* < 0.05). Moreover, although no significant histopathological abnormalities were observed in the examined histological sections, subtle changes were noted. This study evaluated the potential adverse effects of boric acid on bivalves, which are crucial components of the aquatic food chain, utilizing histological and specific physiological parameters following its introduction into aquatic environments. It is anticipated that the findings of this study will contribute to the development of new insights and perspectives regarding the extensive use of boric acid.

## Introductıon

Boron is recognized as an indispensable micronutrient, serving as a trace element vital for the physiological development and growth of organisms [1–3]. It occurs naturally in the environment, forming compounds with other elements, some of which hold considerable commercial significance [4]. For instance, boric acid, identified as a weak monobasic Lewis acid of boron [5], is of notable industrial importance. Its essentiality for plant growth has been long established [6, 7], with uptake occurring through plant consumption and, via water sources, as inorganic boron, subsequently transferring to animal species and humans [8]. Upon its discovery, boron’s significance in plant nutrition was promptly recognized, leading to its widespread application in agricultural practices [3]. The nutritional role of boron in human and animal metabolism gained elucidation in the 1980s [9–11]. In the European Union, boric acid finds authorization as a food additive and preservative in select food items (e.g., caviar) [10], while its utilization in animal nutrition has also been notable in recent years [4]. Boron exerts multifaceted effects on cellular signaling pathways and participates in the formation and modulation of entities involved in numerous biochemical processes. It assumes pivotal roles in the life cycle of highly organized organisms and contributes to various biological phenomena such as cellular structural integrity and enzymatic activities [12]. Furthermore, boron has been implicated in cellular signaling mechanisms, impacting the functionality of diverse organs, including the brain, while also actively modulating immune responses [7, 13, 14]. Beyond its nutritional significance, boron finds extensive utility across diverse domains.

In addition to its utilization in cosmetic, ceramic, and glass industries, boron is frequently favored within industrial sectors such as nuclear technology, materials engineering, and energy production [15]. Consequently, experimental inquiries were launched to explore its impact on clinical health subsequent to the elucidation of its biological significance and role in animal and human metabolic processes [4, 16]. As the usage of boron proliferated, inquiries emerged regarding its potential toxicological implications, juxtaposed against its protective effects on living organisms [17, 18]. The introduction of boron into air, water, or soil ecosystems can be construed as a corollary of its escalating utilization, both naturally and anthropogenically [19, 20]. This surge in boron usage has prompted scrutiny into the toxic ramifications of boric acid [21, 22]. While low concentrations of boron are generally associated with minimal toxicity in soil, water, and living organisms, investigations have delineated its adverse effects at elevated concentrations, leading to its classification within the chemical pesticide group since as early as 1948 [5]. Despite the extensive historical use of boric acid across diverse applications, from medicinal to pesticidal and industrial realms, information pertaining to its potential toxicological effects remains somewhat limited [3]. Although the available literature regarding the toxic effects of boron on animals is currently constrained, prevailing studies predominantly focus on human populations [23, 24] and rodent models [25–28]. Over the past two decades, there has been a growing awareness regarding the acute toxicity of boron on aquatic organisms, stemming from its ingress into aquatic ecosystems via both natural processes and human activities [12]. It is postulated that boron and its derivatives, particularly those introduced into freshwater bodies through agricultural and irrigation wastewater, have the potential to translocate to inland waters and subsequently to marine environments, potentially exerting toxic effects at specific concentration thresholds [29, 30]. The assessment of boron’s potential toxic effects has predominantly focused on fish species [4, 7, 12, 31–38], with limited investigations involving macroinvertebrates [3]. A common consensus derived from these studies is that boron and its derivatives possess the capacity to perturb hormone and lipid metabolism, as well as modulate the activity of numerous enzymes [34, 39, 40]. While the precise extent of these effects on biochemical processes remains incompletely elucidated [33, 34, 41], it is established that boron and its derivatives do not undergo metabolic transformations, with borates introduced into aquatic environments primarily forming boric acid and borate anions [12, 42].

As with any pollutant infiltrating aquatic ecosystems, boric acid harbors the potential to instigate oxidative stress within aquatic organisms via mechanisms involving free radicals and reactive oxygen species (ROS). Aquatic organisms, particularly bivalves, possess the capacity to mount responses to environmental pollutants through a spectrum of immune and antioxidant defense mechanisms [43–45]. Ruditapes species, prominent constituents of coastal ecosystems, bear substantial economic and ecological significance. Their propensity, akin to other bivalves, for pollutant accumulation through filter-feeding renders them valuable focal points in biomonitoring endeavors [46, 47]. The primary objective of this study is to elucidate the deleterious effects of boric acid, a commonly employed substance, on bivalves within aquatic environments. To this end, histopathological alterations and antioxidant responses were evaluated in the digestive gland and gill tissues of *Ruditapes decussatus* specimens subjected to varying concentrations of boric acid over a 96-h period under controlled laboratory conditions.

## Materıal and Methods

### Experimental Design

Samples of *R. decussatus* were procured from Gelibolu Seafood Import Export Industry, Turkey, a local farm. Upon acquisition, the specimens were acclimated in laboratory conditions within aquariums containing 15 L of artificial seawater for a duration of 5 days, equating to approximately 1 L per mussel. Throughout this acclimation period, the artificial seawater was renewed daily, with a complete replacement on the initial two days, followed by a 50% renewal on the subsequent 3rd and 4th days. The experimental design encompassed three replicates, each comprising 10 individuals per concentration level. The acute effects assessment was conducted over a span of 96 h. The concentrations of exposure (0, 0.05, 0.5, and 5 mg/L) were determined based on established doses from prior literature [3, 34]. All experimental groups were provided with aerated environments ensuring requisite water quality parameters. Monitoring of water temperature and dissolved oxygen levels was performed utilizing a YSI MPS 556 probe, while pH values were routinely assessed employing a HANNA C 200 (HI 83200) photometer. Ethical guidelines were rigorously adhered to throughout the experimental procedures.

### Sampling

At the culmination of the exposure duration, ten mussel specimens from each aquarium underwent dissection subsequent to morphological measurements encompassing length, width, and height. Among these samples, five from each experimental group were allocated for histopathological evaluation, while the remaining five were designated for the assessment of antioxidant parameters. Histopathological analyses entailed the examination of mantle, gill, and digestive gland tissues of the mussel specimens. Concurrently, oxidative parameters were ascertained in both gill and digestive gland tissues.

### Oxidative Stress Parameters

Gill and digestive gland tissues were promptly fixed with liquid nitrogen upon collection and subsequently stored at − 80 °C until the commencement of analyses. Prior to analysis, tissue homogenization was performed utilizing a 50 mM phosphate buffer. Oxidative parameters, notably the enzymatic activities of superoxide dismutase (SOD), catalase (CAT), and glutathione-S-transferase (GST), alongside the quantification of lipid peroxidation (MDA), were assessed. To standardize enzyme activities in terms of U.mg.protein^−1^, the protein content within the tissues was quantified employing the Bradford method [48].

SOD activity was evaluated through the reduction of nitroblue tetrazolium (NBT), resulting in the formation of a blue-hued formazan product with maximal absorbance at 550 nm [49, 50]. CAT activity was determined by monitoring alterations in absorbance over a duration of approximately 90 s subsequent to initial tissue measurements [51]. GST activity analysis involved spectrophotometric measurements at 340 nm, taken at distinct time intervals, followed by kinetic computations [52]. Lipid peroxidation, serving as an indicative marker of oxidative damage, was quantified based on the levels of MDA, the terminal product of this oxidative process [53].

### Histopathological Assessment

The mantle, gill, and digestive gland tissues of the mussels underwent fixation in Davidson’s fixative for a duration of 24 h, followed by immersion in a 70% ethanol solution. Subsequent to standard histological preparation protocols, tissue embedding in paraffin blocks facilitated the generation of sections measuring 5 µm in thickness. These sections were then subjected to staining with hematoxylin and eosin, as outlined by Gamble and Wilson [54]. Histopathological alterations were meticulously examined, and visual documentation was facilitated through employment of a CX31 Olympus light microscope, equipped with a digital camera, utilizing DP2-BSW software.

### Data Analysis

The statistical analyses were conducted utilizing SPSS 21.0 software. The normal distribution of the data was assessed employing the Kolmogorov–Smirnov test, while the homogeneity of variances was evaluated using the Levene test. Enzyme analyses and MDA levels underwent comparison via parametric one-way ANOVA and/or non-parametric Kruskal–Wallis tests. Distinct letters or numbers were assigned to denote significant differences among concentrations. The relationship between quantified histological parameters and oxidative measurements was explored through discriminant analysis, ensuring validation for non-linearity and variances. A significance level (α) of 0.05 was adopted for all analyses.

## Results

The morphometric attributes, encompassing length, width, height, and weight measurements of all mussel specimens, are delineated in Table 1. In the study’s inception, a deliberate effort was made to select mussel samples exhibiting comparable lengths and weights, thereby mitigating potential variations stemming from morphological disparities during subsequent analyses.

**Table 1:** The descriptive statistics of morphological measurements (sd: standard deviation) (*n* = 30 per groups)

Concentrations	Length (mm) mean ± sd	Height (mm) mean ± sd	Width (mm) mean ± sd	Weight (mm) mean ± sd
Control	26.87 ± 1.1	40.86 ± 1.6	17.76 ± 0.58	12.55 ± 1.34
0.05 mg/L	27.45 ± 1.1	42.03 ± 2	18.96 ± 0.8	14.2 ± 1.5
0.5 mg/L	27.25 ± 0.87	42.08 ± 1.5	18.76 ± 1.17	14.16 ± 1.75
5 mg/L	26.54 ± 1.2	40.88 ± 1.7	18.7 ± 0.9	13.14 ± 1.46

### Oxidative Stress Parameters

**Fig. 1:**
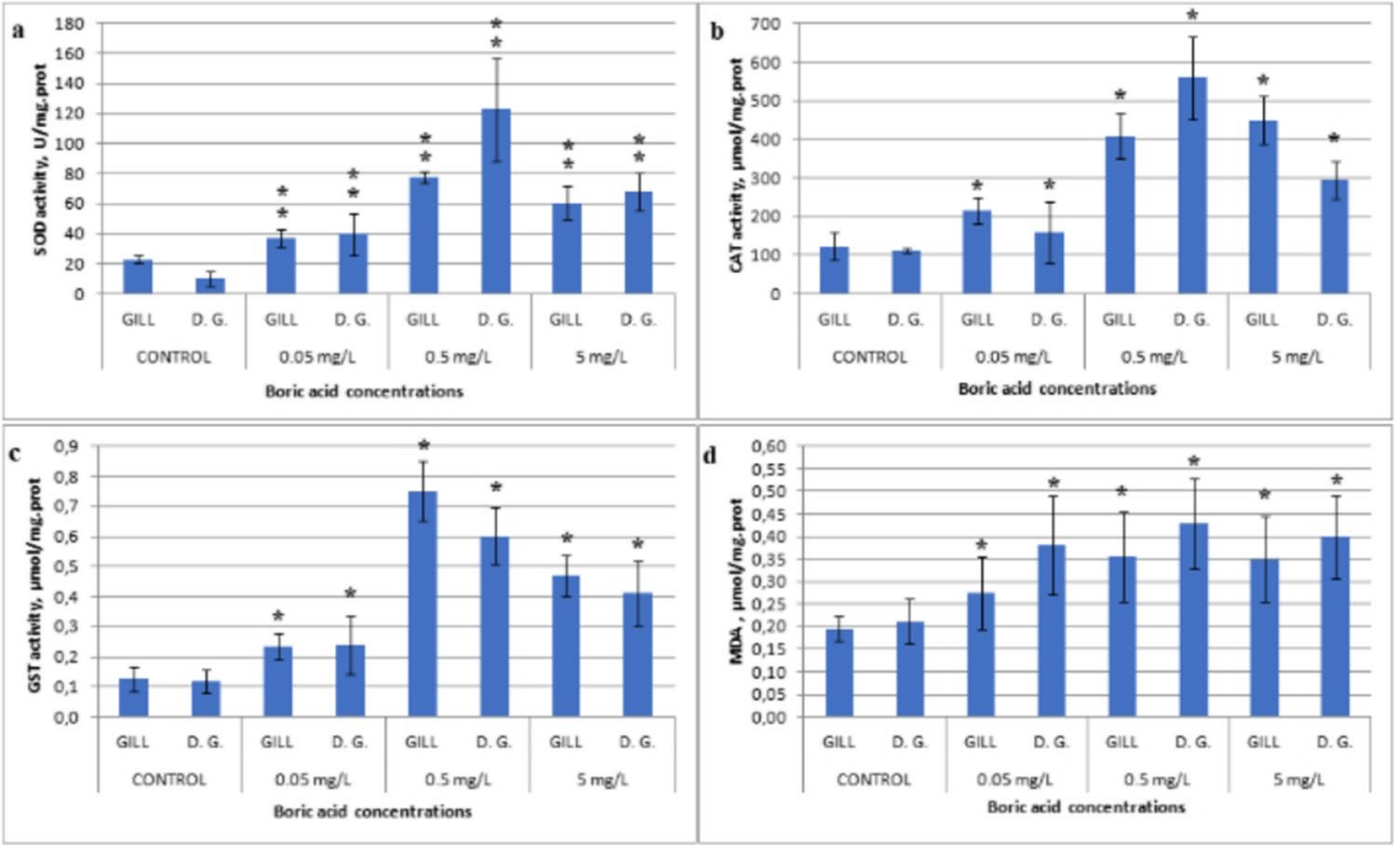
**a** SOD, **b** CAT, **c** GST activities, and **d** MDA level in the gill and digestive gland tissues (D.G: digestive gland) of *R. decussatus* against in vitro boric acid exposures (0, 0.05, 0.5, and 5 mg/L) for 96 h (*the mean difference is significant against the concentrations; **the mean difference is significant against both the concentrations and the tissues; *p* < 0.05)

In the control group, the SOD activity in gill tissue samples, devoid of exposure to any concentration of boric acid, exhibited a range between 20.5 and 26.2 U mg.prot.^−1^, while in the digestive gland tissues, the activity ranged from 4.6 to 15.5 U mg.prot.^−1^. Conversely, in groups exposed to the lowest concentration of 0.05 mg/L boric acid, the SOD values measured in gill tissues ranged from 31.5 to 49.3 U mg.prot.^−1^, whereas in digestive gland tissues, this spanned between 17.5 and 60 U mg.prot.^−1^. Notably, the most elevated SOD enzyme activity values were discerned in digestive gland tissues of specimens subjected to 0.5 mg/L boric acid (168.5 mg.prot.^−1^). Similarly, heightened values were also observed in gill tissues compared to other concentrations. The observed disparity in SOD levels among concentrations exhibited statistical significance (*F* = 63.9, df = 3, *p* < 0.05). Moreover, statistically significant differences were noted in SOD activity among the targeted tissues (*F* = 4.9, df = 1, *p* < 0.05) (refer to Fig. 1a).

The CAT levels in the gill and digestive gland tissues of the control group samples were determined to range between 70 and 157 µmol mg prot.^−1^. Notably, the highest CAT value was observed in the digestive gland tissue of a specimen exposed to 0.5 mg/L boric acid (770 µmol mg prot^−1^), with the mean CAT levels being notably elevated across different tissues within this group (mean 488.9 µmol mg prot.^−1^). Statistical analysis revealed significant differences in CAT levels among concentrations (*F* = 75.1, df = 3, *p* < 0.05) (see Fig. 1b).

Conversely, the GST enzyme levels, representing a phase II detoxification enzyme, were initially quantified at 0.06 µmol mg prot.^−1^ in the gill tissue of the lowest control group. Subsequently, the gill tissue of individuals exposed to 0.5 mg/L boric acid exhibited the highest GST levels (mean 0.12 µmol mg prot.^−1^), followed by those in tissues of specimens exposed to the highest concentration (5 mg/L) (mean 0.09 µmol mg prot. ^−1^), and then the lowest concentration (mean 0.07 µmol mg prot.^−1^), respectively. While fluctuations in GST levels did not attain statistical significance at the tissue level (*F* = 2.05, df = 1, *p* > 0.05), significant differences were discerned across concentrations (*F* = 147.1, df = 3, *p* < 0.05) (refer to Fig. 1c).

Assessed as an index of oxidative damage, lipid peroxidation was manifested through the detection of malondialdehyde (MDA) levels, the end product of this oxidative process. Comparative analysis against the control group revealed elevated MDA levels in both tissues of the exposure groups. Notably, the highest MDA levels were observed in the digestive gland tissue following exposure to 0.5 mg/L boric acid (mean 0.43 µmol mg prot.^−1^), succeeded by levels in the digestive gland tissue under 5 mg/L exposure (mean 0.4 µmol mg prot.^−1^). Statistical examination unveiled significant differences in MDA levels across concentrations (*F* = 10.2, df = 3, *p* < 0.05) and among tissues (*F* = 11.1, df = 1, *p* < 0.05) (refer to Fig. 1d).

### Histopathological Assessment

#### Mantle

**Fig. 2:**
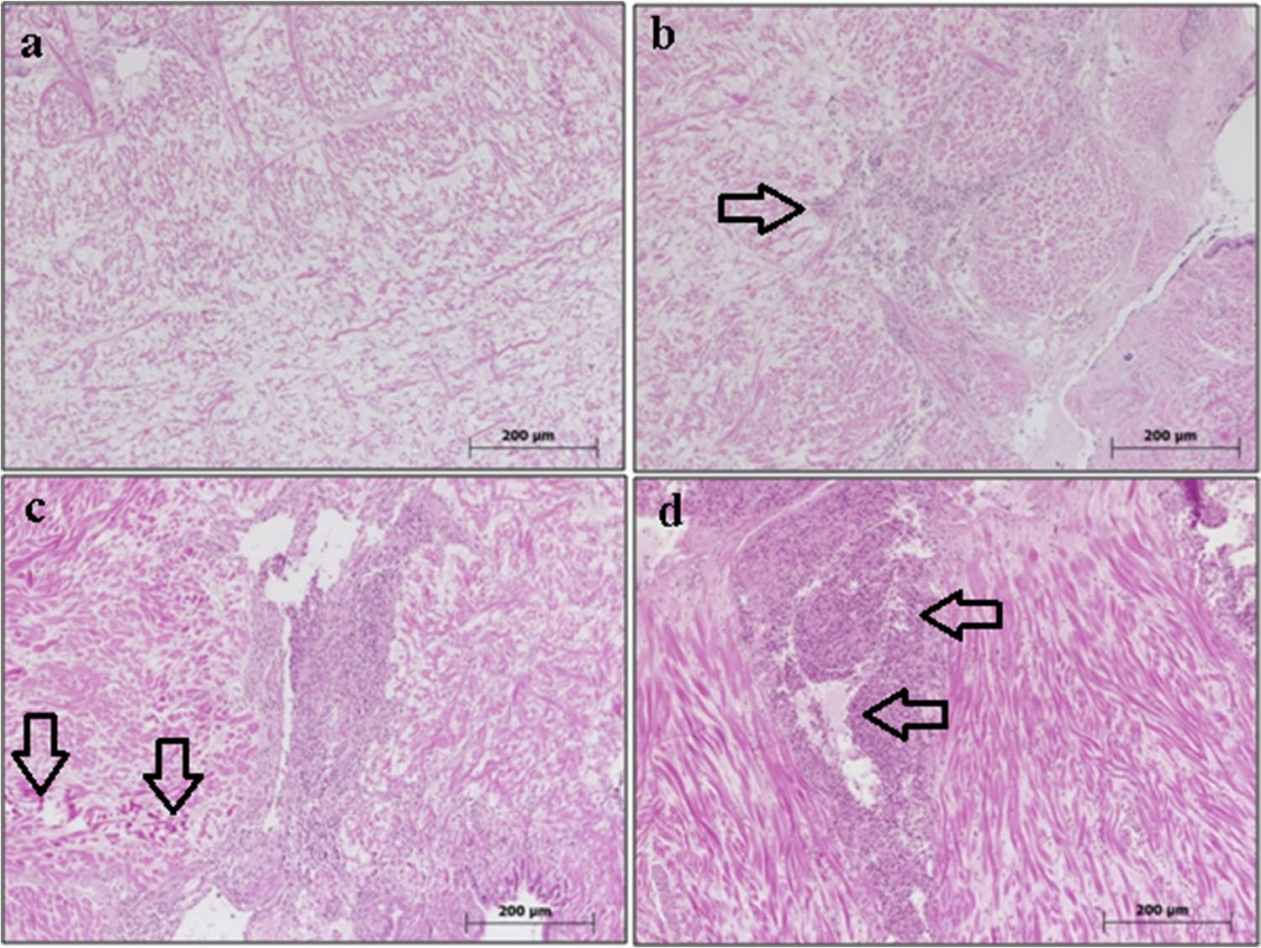
Mantle sections of *R. decussatus*. **a** Control (0 mg/L), **b** 0.05 mg/L, **c** 0.5 mg/L, and **d** 5 mg/L boric acid. Hemositic infiltrations indicated by arrows, H&E

No histopathological aberrations were evident in the mantle sections of the control group (refer to Fig. 2a). Nonetheless, hemositic infiltrations were discerned in the mantle sections of the cohort exposed to 0.05 mg/L boric acid (refer to Fig. 2b). Subsequent observations indicated the pervasiveness of this finding across other doses (0.5 and 5 mg/L) throughout the study (refer to Fig. 2c, d).

#### Gill

**Fig. 3:**
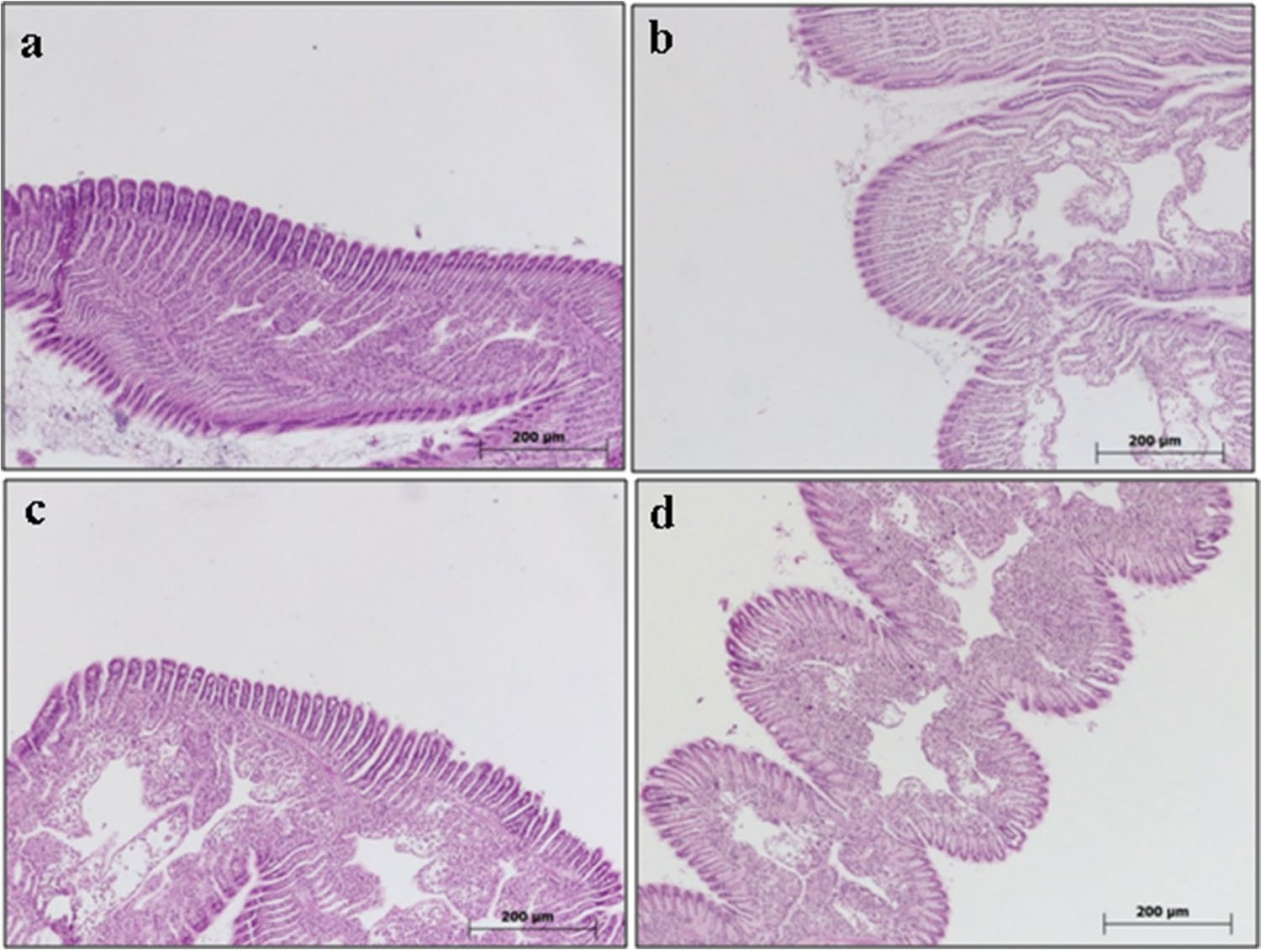
Gill sections of *R. decussatus*. **a** Control (0 mg/L), **b** 0.05 mg/L, **c** 0.5 mg/L, and **d** 5 mg/L boric acid. H&E

The gill sections of mussels in the control group exhibited a histologically normal appearance (refer to Fig. 3a). Notably, no histopathological anomalies were noted across any of the administered doses (refer to Fig. 3b–d).

#### Digestive Gland

**Fig. 4:**
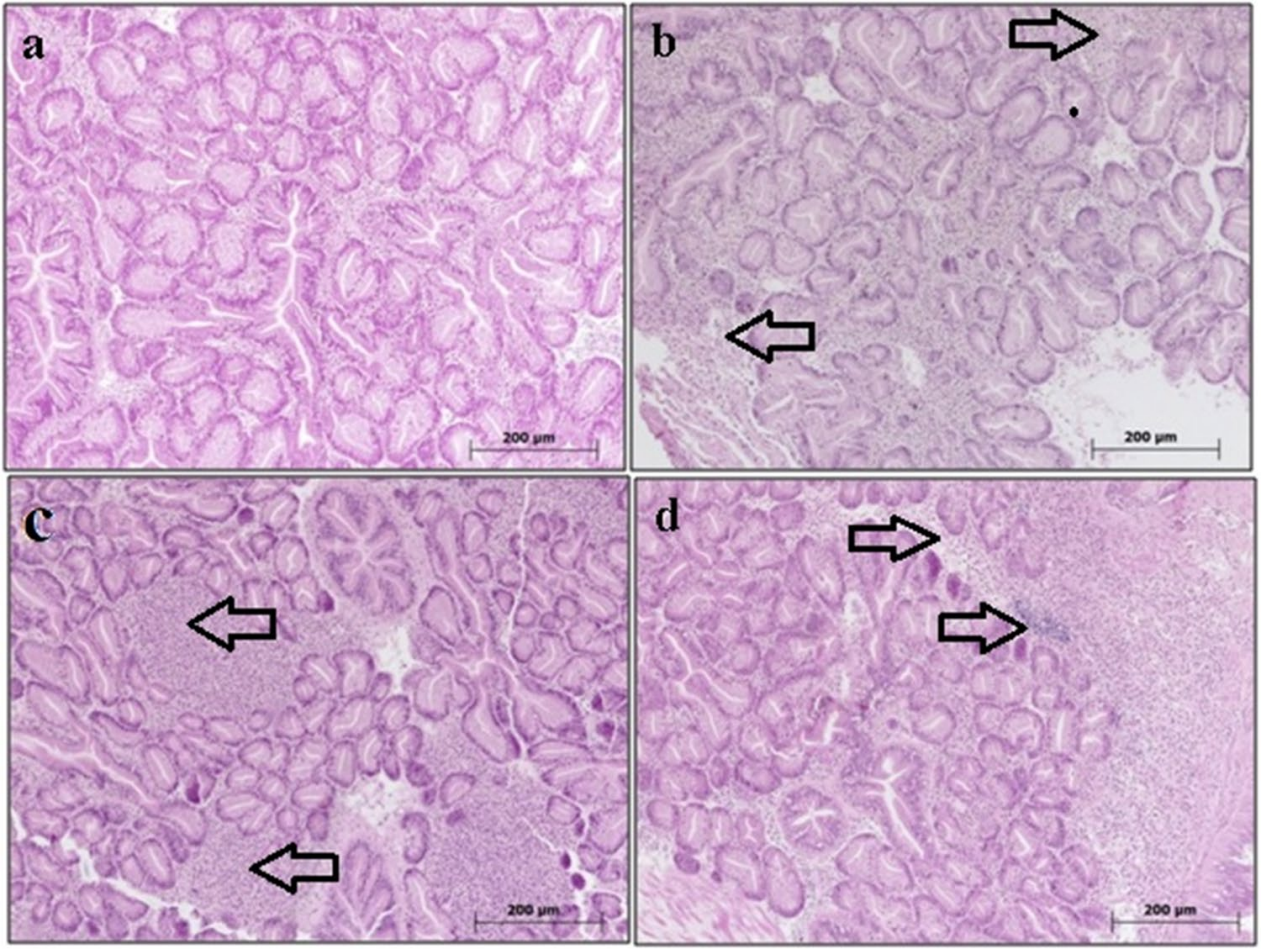
Digestive gland sections of *R. decussatus*. **a** Control (0 mg/L), **b** 0.05 mg/L, **c** 0.5 mg/L, and **d** 5 mg/L boric acid. Hemositic infiltrations indicated by arrows, H&E

The digestive gland sections of mussels within the control group displayed histologically normal tubular structures (refer to Fig. 4a). However, specimens exposed to 0.05 mg/L of boric acid exhibited localized hemositic infiltrations (refer to Fig. 4b). Notably, the pervasiveness of hemositic infiltrations increased notably in the group subjected to 0.5 mg/L boric acid exposure (refer to Fig. 4c). Furthermore, specimens exposed to 5 mg/L of boric acid showcased pronounced hemositic infiltrations along with epithelial deformations within the digestive gland tubules (refer to Fig. 4d).

## Discussion

Boric acid, one of the twelve naturally occurring boron-containing compounds [55], is widely employed for its therapeutic attributes in addressing inflammatory conditions [56]. Its historical use as a pesticide in agricultural practices spans many years [21, 57], and reports also indicate its antifungal or fungistatic properties [58–60]. Given its application as an inorganic chemical insecticide, studies have revealed that boric acid can disrupt specific physiological and biochemical processes in non-target organisms [5, 61, 62].

In investigations spanning both vertebrate and invertebrate taxa, boric acid has been observed to manifest among the lowest degrees of bioaccumulation and associated potential toxicities [59]. Studies concerning boron toxicity predominantly emphasize developmental biology [24]. Various inquiries targeting diverse fly species [63], assorted insect taxa [64, 65], and even human subjects [66] have delineated adverse outcomes linked to boron and its derivatives across distinct developmental stages. Illustrating aquatic ecosystems, observations have indicated variances in the growth of certain fish species correlating with boron concentrations [37, 38].

When considering the potential impact of substances introduced into aquatic ecosystems, it becomes evident that they may manifest toxic effects over time owing to bioaccumulation, thereby disrupting the ecosystem’s functionality and adversely affecting organisms across various trophic levels [67]. It is noteworthy that the manifestation of toxicity in aquatic organisms can exhibit variability contingent upon the specific species involved [12, 68]. Concurrently, research endeavors assessing the toxicity of boron and its derivatives in aquatic organisms, in conjunction with growth factors, have been documented. In the realm of acute exposures, lethal concentrations (LC_50_) have been delineated for boron and its derivatives across diverse fish species. These concentrations were elucidated as 74 mg/L for dab (*Limanda limanda*) [69], 43 mg/L for coho salmon (*Oncorhynchus kisutch*) [70], 979 mg/L for mosquito fish (*Gambusia affinis*) [71], 108–252 mg/L for flounder (*Paralichthys olivaceus*), and 97–172 mg/L for sea bream (*Parus major*) [18].

In a prior investigation, it was observed that boron derivative concentrations below 10 mg/L did not manifest toxic effects on trout species [2]. Leveraging this observation, the current study aimed to evaluate the histological ramifications and quantifiable physiological responses induced by borax acid, a widely utilized substance spanning diverse domains, on specimens of *R. decussatus*, a bivalve species integral to human consumption. Employing a meticulously devised experimental framework, artificial seawater was meticulously concocted, facilitating a 96-h exposure of the samples to borax acid. Histological assessments unveiled an absence of cellular alterations in specimens exposed to concentrations below 5 mg/L, contrasting starkly with pronounced signs of hemositic infiltration and epithelial deformation observed at the highest concentration. The escalating prevalence of histological irregularities in tandem with concentration corroborates prior research illustrating the toxicity profile of boron and its derivatives [22, 72–74].

Numerous studies have been conducted to evaluate the extent of oxidative damage and genotoxicity induced by boron and its derivatives. Particularly within investigations involving mammalian groups, it has been documented that boron and its derivatives elicit increases in antioxidant levels, while the ensuing damage lacks genotoxicity [41, 75–81]. Nonetheless, despite the myriad of evaluations undertaken, definitive establishment of the effect of boron and its derivatives on antioxidants remains inconclusive [4]. In an effort to elucidate the antioxidant defense system concerning potential physiological or pathological conditions that may ensue in mussel samples subsequent to exposure, levels of SOD, CAT, and GST enzymes were scrutinized, alongside the assessment of LPO quantity to gauge oxidative damage. It is envisaged that concomitant with the escalation of reactive oxygen species (ROS) upon exposure, the delicate balance of antioxidants will be disrupted [82]. Furthermore, the stress response that mussels may exhibit to varying concentrations of boric acid is favored due to its propensity to perturb normal body homeostasis, culminating in an array of biochemical, physiological, and behavioral alterations.

At concentrations ranging up to a maximum of 5 mg/L, evidence suggests the potential initiation of oxidative damage in two distinct tissue types within the samples. Following acute exposure to boric acid, notably heightened antioxidant levels were discerned at a concentration of 0.5 mg/L, denoting a moderate concentration level. Noteworthy is the observation that the pinnacle levels of enzymes catalyzing Phase I reactions, such as SOD and CAT, were predominantly present in the digestive gland tissue. Conversely, the GST enzyme, instrumental in Phase II reactions, exhibited its highest values within the gill tissue. This observed variance between tissue types may be correlated with the gill tissue’s precedent exposure to boric acid, which appears to be both earlier and more extensive.

The prompt response exhibited by the SOD enzyme within this context can be attributed to its capacity to uphold the primary line of defense without necessitating an increase in the prevailing metabolic energy reservoirs of mussels [83]. Analogously rapid SOD responses have been documented in exposure investigations encompassing diverse mussel species subsequent to pollutant exposure [84–86]. Moreover, varying concentrations of boron compounds have been shown to induce heightened SOD levels [75]. Nevertheless, contrary to initial expectations, this study revealed that SOD levels did not exhibit a linear increment with escalating concentrations of boric acid; rather, a tendency towards reduction was noted at higher concentrations, aligning with findings in extant literature [4, 77]. It could be posited that this declining trend might be associated with the depletion of detoxification mechanisms [87, 88].

The concentrations of CAT and GST displayed a progressive increase from the lowest to the moderate levels, mirroring the trend observed in SOD activity, yet exhibited a decline to lower levels at the highest concentration. The elevation in CAT activity can be attributed to its defensive role against oxygen radicals generated during exposure to boric acid [4]. Conversely, the heightened catalase (CAT) activity in response to increased hydrogen peroxide (H_2_O_2_) levels in both tissue types of boric acid-exposed specimens implies the presence of exposure-induced redox imbalance [89]. Considering the cooperative action of CAT and GST enzymes against oxidative stress in both the gill and digestive gland tissues, the concentration-based results remain consistent. However, notably, disparate increases in enzyme activities across different organs were particularly evident at the 0.5 mg/L exposure level. This finding at the 0.5 mg/L boric acid exposure strengthens the notion that the initial response in gill tissue entails GST activity, whereas in the digestive gland tissue, it involves CAT induction under the same exposure conditions [90]. These increments in enzyme levels substantiate the occurrence of oxidative stress consequent to boric acid exposure in mussels, stemming from an imbalance in pro-/antioxidant metabolism, thus supporting the concordance of oxidative effects associated with boric acid toxicity with prior research [4, 34, 41, 75–81].

When faced with a contaminant, the structural integrity of cell membranes can be compromised, leading to the deactivation of membrane-associated enzymes and receptors, a phenomenon termed lipid peroxidation [91]. Levels of lipid peroxidation serve as specific indicators of the activity status of antioxidant systems [92–94]. In the current investigation, the probability of encountering a moderate oxidative stress scenario resulting from the elevation in tissue MDA levels attributed to boric acid exposure has been reinforced. Measurements taken in the digestive gland tissues exhibited significantly higher values across all three concentrations. The highest LPO values were documented at a concentration of 0.5 mg/L. Prior studies have also documented escalated lipid peroxidation phenomena in aquatic organisms following exposure to pollutants [95–103].

This study aimed to assess the toxic potential of boric acid, which is extensively utilized across various sectors, in *R. decussatus*, an integral species in the food chain due to its filter-feeding behavior. The investigation focused on elucidating the potential adverse effects of boric acid by examining histopathological changes and antioxidant responses upon its introduction into aquatic ecosystems. The findings revealed notable physiological and specific histological alterations in mussels as a result of boric acid exposure. Considering its industrial and agricultural utility, the study underscores the importance of judicious boric acid usage to mitigate potential harms and ecological ramifications.

## Data Availability

The datasets generated during and/or analyzed during the current study are not publicly available but are available from the corresponding author on reasonable request.
